# Pulsed Electric Field Extraction of α and β-Acids From Pellets of *Humulus lupulus* (Hop)

**DOI:** 10.3389/fbioe.2020.00297

**Published:** 2020-04-17

**Authors:** George Ntourtoglou, Evangelia Anastasia Tsapou, Fotini Drosou, Eleni Bozinou, Stavros Lalas, Panagiotis Tataridis, Vassilis Dourtoglou

**Affiliations:** ^1^Department of Wine, Vine and Beverage Sciences, University of West Attica, Athens, Greece; ^2^Department of Food Science and Nutrition, University of Thessaly, Karditsa, Greece

**Keywords:** hops, pulsed electric field, α-acids, β-acids, extraction, SPE

## Abstract

This paper investigates the process of extracting hop pellets (hops) utilizing the pulsed electric field (PEF) technique and the contrasting effects of the technique between two distinct hop varieties (one bitter and one aromatic). The effect of PEF on the extraction was evaluated by measuring the concentration of α-acids and β-acids (humulones and lupulones). Regarding the aromatic character, the hop’s volatile caryophyllene, humulene and β-myrcene were analyzed both with and without employing the PEF treatment. In order to analyze the acids and the volatile fraction, the analytical method of UV–vis spectrophotometry was applied followed by gas chromatography coupled with mass spectrometry. For the second technique, the extracts were previously purified through a Graphitized Carbon Black syringe for Solid Phase Extraction. The results revealed that PEF had a positive impact on the alpha acids of bitter hops by increasing the extraction rate of these acids by 20%, while the volatiles demonstrated an increase of 5.6 and 7.4% for humulene and caryophyllene, respectively. Concerning the aromatic variety of hops, the PEF treatment appeared to have no noteworthy effects.

## Introduction

Hop pellets are renowned for contributing to the bitterness of the taste and the enrichment of aroma in beer. They come from the plant *Humulus lupulus* and, specifically, from its female cone. The genus is represented by two species; the Humulus, the common hops (*H. lupulus* L.), and the Japanese hops (*H. japonicus* Sieb. and Zuce.). The Humulus genus particularly, belongs to the family of the Cannabinaceae (Steinhaus and Schieberle, 2000). Hops complement beer in a complex way due to their chemical composition, which varies depending on the variety used, the cultivation techniques and the extraction that occurs during the processing of the beer. In air-dried hop cones, water accounts for 10%, total resins for 15%, essential oils 0.5–2%, tannins 4%, monosaccharides 2%, proteins 15%, ash 8% and cellulose 43% (Stevens, 1967). Additionally, it is remarkable to mention that hops have been used in folk medicine in the past since they possess a broad spectrum of medico-pharmaceutical properties. The hop pellets are financially exploited primarily by the beer industry.

Hop resins include hard resins, soft resins and uncharacterized resins. Hard resins make up the part of the total resins which is insoluble in low boiling paraffinic solvents. Soft resins contrastively, are the fraction of total resins soluble in low boiling paraffinic hydrocarbons and mainly include the α-acids and the β-acids. The α-acids consist of humulones, cohumulones and adhumulones while the β-acids include lupulones, colupulones and adlupulones (Stevens, 1967). There is also another part of the resins which are uncharacterized. This fraction is the portion of the soft resin remaining after precipitation of α-acids with lead acetate and crystallization of β-acids (Stevens, 1967; Kunze, 2004).

Another important constituent of the hop flower, is the essential oils located within the hop cone. This fraction is also known as “hop oil” and is mainly composed by the volatile aromatic compounds. The total oil content depends on the variety of hop and varies between 0.1 and 2.0% by dry weight (Stevens, 1967). In this fraction more than 400 hop flavor components have been identified in majority monoterpenes (C_10_) and sesquiterpenes (C_15_). The main volatiles in hops cultivars are myrcene, α-humulene and β-caryophyllene, which account for 80% (Rettberg et al., 2018). Myrcene varies from variety to variety and can contain from 10 to 72% of the “hop oil”. This compound bestows the green fresh note with resinous aspects (Steinhaus and Schieberle, 2000; Nance et al., 2011). Myrcene’s oxidation forms many terpenoids, such as linalool, geraniol, citral, a-terpineol and carvone, known for their augmenting effects on the aroma (Dieckmann and Palamand, 1974; Rettberg et al., 2018). With respect to α-humulene (15–42% of the essential oil of hops) and β-caryophyllene (2.8–18.2% of the essential oil), they are known for their woody and spicy odor (Peacock and Deinzer, 1981; Nickerson and Van Engel, 1992; Peacock and McCarty, 1992; Eyres and Dufour, 2009).

Conventional extraction methods require extended extraction times, high purity solvents, often offer low extraction selectivity and, finally, in some cases are responsible for the thermal decomposition of sensitive compounds (Bozinou et al., 2019). For the above reasons, new extraction techniques have been introduced. These methods include ultrasonic waves (Hossain et al., 2014; Bimakr et al., 2017), gamma irradiation (Gyawali et al., 2006; Pinela et al., 2014) and electric fields, including the pulsed electric field (PEF) (Delsart et al., 2012; Zhang et al., 2012). These methods are already applied to other crops of commercial interest such as grapes, onions, potatoes, etc.

New technologies, such as PEFs and high voltage electric discharges (HVED), have been proposed for microbial inactivation of food liquids (Delsart et al., 2015), for the extraction of compounds from Chardonnay grapes (Grimi et al., 2009) or other fruits, such as apples (Grimi et al., 2011). HVEDs have also been proposed for the extraction of polyphenols and other compounds with antioxidant activity (Boussetta et al., 2009; Boussetta et al., 2011).

The application of PEF has principally been used as a non-thermal treatment of liquid foods aiming to the inactivation of microorganisms (Grahl and Markl, 1996; Alvarez et al., 2003). The microbial inactivation is a function of food composition which depends on the composition of the solution and the electrical parameters (Heinz et al., 2003; Touya, 2005; Vorobiev and Lebovka, 2010). Other researchers have introduced electric field treatment for the acceleration of aging of young wine thanks to the extraction of flavor compounds from wood (Zeng et al., 2008; Drosou et al., 2017).

The disruption of the cell membrane due to electroporation is caused by the high intensity of the fields induced by PEF. This disturbance of the architectural structure of the membrane and the disorganization of the integrity of microbial or plant cells, lead to complex phenomena such as cell lysis or the fusion of protoplasts. When the transmembrane potential exceeds a critical value, generally around 0.8 to 1 V (Zimmermann, 1986), pore formation occurs in the cell membrane and certain metabolites diffuse in the extracellular medium. This state can be transient and reversible if the applied field remains below a certain level (Cukjati et al., 2007). On the other hand, the electropermeabilization of cells must be irreversible when the objective is the inactivation of microbial cells.

The PEF treatment system is not a simple device and consists of a high voltage source, in some cases a capacitor bank, a switch and the treatment chamber. The PEF treatment chamber comprises two or more electrodes, filled with the material to be treated and it is constructed so that the electric field acting on the mass of the product to be treated is as homogeneous as possible (Maged and Ayman, 2012).

Hops are the most complex and costly raw material used in brewing. Of all the herbs that have been used to flavor and preserve beer over the ages, only the hop (*H. lupulus* L.) is now regarded as an essential raw material in brewing throughout the world.

In 2017, 148,603 tons of hops were produced worldwide (FAO). The majority was produced in the United States, with a total of 47,000 tons. Considerable amounts were also produced in Ethiopia and Germany yielding up to 38,000 and 32,000 tons, respectively. The estimated needs for α-acids are calculated up to 8,000 tons, and the average price is valued approximately at 8 United States $ per kg. Demands for alpha acids are estimated on the basis that an average of 4.1 g is needed per hectoliter of beer (European Commission). Hop content varies depending on the type of beer, particularly considering its bitterness, and the variety of hop used. Hop content displays a steady decline in percentage annually (it still stood at 6.3 g alpha per hectoliter in 1995) due to the consumers’ growing preference for less bitter beers, and the technological progress that this preference has brought about. Different brewing techniques have been developed to enhance the extraction of volatiles and acids in hopped beers. The most significant contribution of hops to beer flavoring is that of the so-called soft resins, principally the alpha acids (also known as humulones), which are ultimately responsible for the characteristic bitterness in the taste.

Analytically, the aroma of hops and the flavor of hoppy beers cannot be measured by the quantification of a single odorant; moreover, the selection of several key compounds or a comprehensive characterization (profiling) is of great importance. Analysis of hops and beer is challenging.

This study determines the effect of PEF treatment on two hop varieties for the extraction of bitter acids and volatiles. No previous studies have been published on this field to the extent of our knowledge. Additionally, research on the divergent effects of the treatment on two separate hop varieties (bitter and aromatic), was carried out.

## Materials and Methods

### Plant Material

The two different varieties of hop cones (pelletized) used in this study were purchased by the Macedonian Thrace Brewery S.A. (Athens, Greece). The first variety was bitter, known for its high content in “bitter” acids. The second variety was aromatic, known for its high quality of essential oils. The characteristics of the two varieties were determined (using the methods described below).

#### Moisture Content Determination

For the determination of the dry matter content of hops, an established method regarding the moisture content of hops and hop products by European Brewery Convention, 2006 (EBC, 7.2, 1998) was employed. After being weighted, the samples were dried in a vacuum oven in 85°C for 6 h. The moisture percentage was determined according to the following equation: Moisture in, hops was calculated as:% = $\frac{\mathrm{loss}\,\mathrm{in}\,w\,t\times 100}{w\,t\,\mathrm{of}\,\mathrm{sample}}$ (loss in wt × 100)/(wt of sample).

#### Hop Storage Index (HSI)

The determination of HSI was carried out according to the American Society of Brewing Chemists and specifically the Method of Analysis HOPS-6.A, where, the oxidative decrease in both α- and β-acids content during storage is determined by the progressive increase in the ratio of absorbance at 275–325 nm. Such loss in α- and β-acids and increase in the hop storage index (HSI) ratios may reflect unfavorably on the utility and quality of the hops.

The HSI was calculated on a ratio of absorbance at 275 nm (A_275_) to the absorbance at 325 nm (A_325_) after PEF treatment and compared to the same ratio without PEF (control).

### Chemicals

The dichloromethane, chloroform, sodium chloride, ethyl acetate, methanol, N-pentane, anhydrous sodium sulfate and 2-octanol used were purchased from the Chem Lab (Zedelgem, Belgium).

### PEF Equipment

**FIGURE 1:**
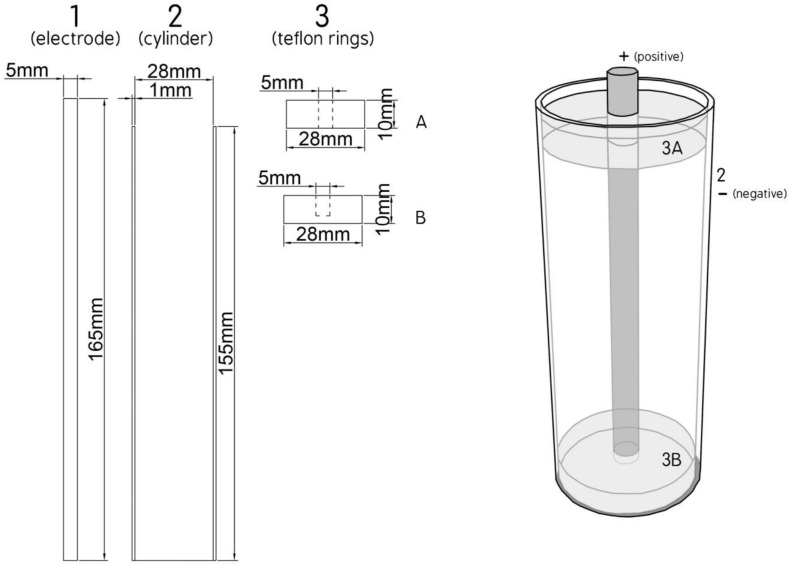
View of treatment chamber.

The PEF equipment used was provided by Val-Electronic (Athens, Greece), and included the static bench scale system, reported previously (Bozinou et al., 2019), accompanied by another high voltage power generator (from Eisco, India). The model of the batch processing chamber (TC) was adapted from a design of cylinder type electrodes (Ohshima and Sato, 2004) and consists of a coaxial stainless steel electrode [5 mm in diameter and 165 mm high, Figure 1(1)] placed inside a bronze cylinder [1 mm thick, 155 mm high and 30 mm outer diameter Figure 1(2)] with a closed flat bottom. In this cylinder are placed two teflon rings (28 mm diameter and 10 mm thick Figure 1(3), one at the bottom Figure 1(3B) and another at the top Figure 1(3A) with a hole in the middle to pass the electrode which serve to isolate the electrode of the outer bronze cylinder (Figure 1).

The electric field strength *E* is evaluated as *E* = U/d, where “U” is the applied voltage and “*d*” is the distance between the electrode and the bronze cylinder (*d* = 12.4 mm).

For each case, the treatment was calculated as:

t=(ti+tp)×P

ti = pulse duration (sec) tp = pause time (sec)

*P* = number of pulses.

For GC-MS analysis the extraction solvent was methanol. For UV–vis analysis the extraction solvents were methanol, toluene and water. For the capacitance measurement the solvents were methanol, water and ethanol. For the extraction method with water the electric field strength was *E* = 2.42 kV/cm, *t* = 30 min (ti = 1 μs, tp = 1 s, 1800 pulses), while for all the others solvents the treatment conditions were *E* = 1.13 kV/cm, *t* = 30 min (ti = 1 μs, tp = 1 s, 1800 pulses).

For the combination “treatment chamber- sample” there was no dielectric breakdown until the electric field strength of 2.5 kV/cm for 56 μF capacitance of the discharge capacitor. On these grounds, 1.15 to 2.5 kV/cm were used during this work. In order to select the number of pulses, UV–vis determinations were utilized to quantify the difference between treated samples and controls. Absorbance was measured every 900 pulses. Accordingly, the number of pulses selected was 1800. The pulse width was approximately 1 μs and the frequency of the pulse was 1 Hz. Treatment time was 0.75 ms. The temperature raise caused by the treatment was negligible (<1°C).

### Sample Preparation for PEF and Control Treatments

Around 6 g of hop pellets were grounded to a fine powder using a grinding bowl. A 2.5 g amount of this powder was weighed in a Schott Duran laboratory bottle (100 mL) with Teflon-lined screw cap, and then, 50 mL of methanol was added (same procedure for the other solvents). For the control, another identical sample was prepared and both they left at 25°C for 30 min. Then, one sample was transferred to the treatment cell for PEF and the other was used for control. After the treatment (30 min) both samples were gravity filtered to remove the plant material and the filtrate (hop extract) was transferred into a vial (20 mL) for analysis as described below.

For the experiment with hydrated hop pellets, an additional step was added for the two samples (treated and control) which consist of a 30-min hydration in HPLC water before treatment in methanol or water.

For the evaluation of the treatment time on the extractability of the acids, the extraction medium was methanol. Treatments of 15, 30, 45, and 60 min (increments of 900 pulses) were performed. And at the end of each time, the treated hop pellets were filtered and processed as described above. The same procedure was also carried out for the control sample.

In all treatments, care must be taken to keep the temperature below the boiling point of the solvent used.

### *α*- and *β*-Acids Determination Using UV–Vis Spectra

The method used was adapted from Alderton et al. (1954) and Egts et al. (2012). Specifically, in a 25 mL volumetric flask, 50 μL of the filtrate was added to a methanolic solution of NaOH (0.5 mL of 6M NaOH in 250 mL of methanol) and the complete spectrum (520 to 210 nm) was recorded against a solution of methanol in methanolic NaOH (50 μL:25 mL) as a blanc. The formulas used to find α-acid, β-acid and a third component (comp 3) are the following:

$${\text{A}}_{355}=31.8\,C_{\alpha }+46.0\,{\text{C}}_{\beta }+1.0\,{\text{C}}_{\mathrm{comp3}}$$

$${\text{A}}_{325}=38.1\,C_{\alpha }+33.1\,C_{\beta }+1.5\,C_{\mathrm{comp3}}$$

$${\text{A}}_{275}=9.0\,{\text{C}}_{\alpha }+3.7\,C_{\beta }+3.1\,C_{\mathrm{comp3}}$$

where A_355_, A_325_, and A_275_ stand for the absorbance of the three analytical wavelengths and C_α_, C_β_, and C_comp3_ stand for the concentrations (in mg/L) of the α-acids, β-acids, and the third component, respectively (Egts et al., 2012).

### α-Acids, β-Acids and Terpenes, Determination Using GC-MS

Prior to GC-MS analysis, the hop extracts were purified by applying a solid phase extraction treatment (SPE) using a graphite carbon black syringe (GCB). The syringe was first washed with 10 mL of dichloromethane (DCM) and then conditioned with 10 mL of methanol and 10 mL of deionized water under vacuum to the point of complete dryness. After that, 5 mL of methanolic hop extract was added with 3 mL of distilled water to the GCB syringe. The vacuum was then adjusted to give a flow of 10 mL/min and the cartridges were dried under full vacuum for 10 min. When the cartridges were dried, they were eluted with 5 mL ethyl acetate and 5 mL DCM. The eluents were collected, then dried over sodium sulfate and filtered before adding 50 μL of the internal standard (2-octanol 2500 ppm diluted in the pentane). The sample was then concentrated into a flash evaporator to 1 mL and 1 μL of the sample was injected to the GC-MS.

### Capacitance of the Treatment Chamber

In order to measure the capacitance of the treatment chamber, the chamber was consecutively filled with the materials used in the experiments. To achieve a correct capacitance measurement, the treatment chamber must be electrically discharged. For each of the materials the value of the capacitance was measured with a digital capacitance meter (ProsKit MT-5110, Prokit’s Industries Co. Ltd., Taiwan) with precision ± 0.5%.

### Gas Chromatography/Mass Spectrometry Analysis

The instrumentation, the column and the conditions of GC-MS used were previously described by Drosou et al. (2017).

### DPPH^∙^ Assay

The antioxidant activity of hop extracts was determined using the DPPH^∙^ assay. A slightly modified method of Blois (1958) was adopted. At first, the samples were properly diluted in methanol or ethanol (1:10). An aliquot of 0.1 mL of each diluted extract was added to 3.9 mL of DPPH^∙^ radical solution (0.0029 g/100 mL methanol) and the solution was then vortexed. After 20 min of remaining in the darkness, the absorbance of each mixture was measured at 515 nm. Pure methanol with the DPPH^∙^ radical was used as control. All samples were prepared in triplicate. Percentage of inhibition of DPPH^∙^ radical *I* (%) of each hop extract was calculated according to the following equation:

$$I\left(\%\right)=\left[\frac{A_{\text{blank}}-A_{\text{sample}}}{A_{\text{blank}}}\right]\times 100,$$

where A_blank_ stands for the absorbance of DPPH^∙^ with methanol instead of sample and A_sample_ is the absorbance of DPPH^∙^ after the reaction with hop extracts.

### Statistical Analysis

**FIGURE 2:**
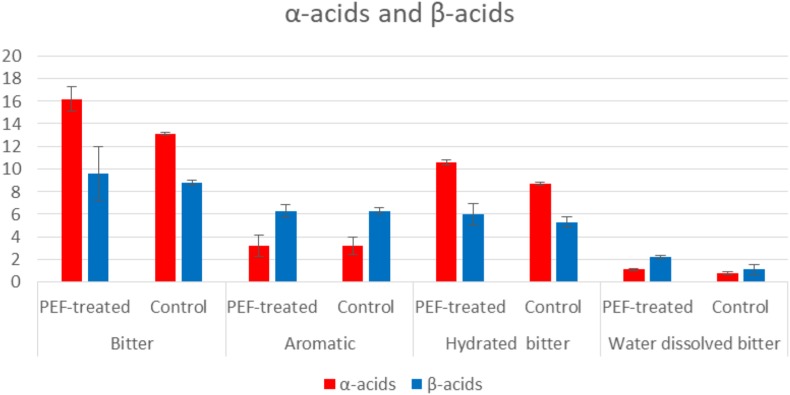
% extraction of α-acids and β-acids determined by UV–vis spectrophotometer.

Results are displayed as means of triplicate determinations. Statistical analysis was carried out using the Excel 2013 (Microsoft, United States) software. Standard deviation for the concentrations of α- and β-acids was calculated and presented in Tables 5, 6 and in Figure 2.

### Risk Assessment

Treatments in organic solvents should be done with caution. In general, in the absence of water or even in binary water-flammable organic solvent systems, the flash point of the solvent or mixture should be taken into account; the temperature should not be increased, preferably at room temperature and the treatment should be carried out in closed systems. Avoid electrical sparks around the treatment chamber.

## Results and Discussion

### Hop Storage Index (HSI)

The physical and chemical values of the two varieties were determined prior to the PEF treatment in order to acquire knowledge on the composition of hop samples. As it was reported by Roberts (2016), HIS is a measure of the degradation and can be used to quantify the losses of α-acids and β-acids during treatment. As it is shown in Table 1, HIS values (0.3–0.4) in bitter and aromatic hop are low, indicating a fresh raw material, as stated by Van Holle et al. (2017). Following PEF treatment, extracts were subjected to an analysis based on their UV–vis spectra.

**TABLE 1:** Hop moisture, hop storage index (HSI), α- and β-acids content and α-acids losses.

	**Hop variety**	
	**Aromatic**	**Bitter**
Moisture (%)	9.47	9.06
HSI	0.43	0.29
α-acids (%)	2.30	10.80
β-acids (%)	3.20	8.40
α-acids losses (%)	105.36	91.87

As it can be observed (Table 1) the bitter hop variety had a 249% higher concentration of total acids compared to the aromatic one. The α-acids are the precursors of iso-α-acids which are formed in the boiling wort resulting in the bitterness of beer. Specifically, the α-acids had 370% higher concentration compared to the aromatic, while for β-acids the concentration was 162%. The results above, partially clarify the difference observed in the extractability using PEF. After PEF treatment, the HSI level varied with the extraction media. In samples treated with methanol the value was 0.58, while for those treated with water, it was 0.87 (0.34 and 1.01 for the control sample, respectively) (Table 2). It appears quite evidently, that following the processes of extraction and treatment with PEF, there is an increase in HSI in both bitter and aromatic varieties but in insignificantly low values indicating that PEF has not any deleterious effects in the raw material. These values of course, are merely results of comparison between hop samples and not between different extraction media, in which differences of solubility drastically influence the final result.

**TABLE 2:** Analysis of spectra from UV–vis.

		**Bitter, methanol extracted**		**Aromatic, methanol extracted**		**Bitter, hydrated and methanol extracted**		**Bitter, hydrated and water extracted**	
		**PEF-treated**	**Control**	**PEF-treated**	**Control**	**PEF-treated**	**Control**	**PEF-treated**	**Control**
Absorbance in nm	275	0.534	0.396	0.246	0.242	0.345	0.266	0.161	0.104
	325	0.918	1.153	0.425	0.422	0.712	0.589	0.185	0.102
	355	0.903	1.139	0.455	0.452	0.686	0.575	0.183	0.097
Acids	α-acids	16.2%	13.1%	3.2%	3.2%	10.6%	8.7%	1.1%	0.8%
	β-acids	9.6%	8.8%	6.3%	6.3%	6%	5.3%	2.2%	1.1%
Increase with	α-acids	24%		0%		21%		100%	
PEF treated for	β-acids	9%		1%		14%		120%	
HSI		0.58	0.34	0.57	0.57	0.48	0.44	0.87	1.01
									

### Effect of PEF on the Extractability of *α*- and *β*-Acids

**FIGURE 3:**
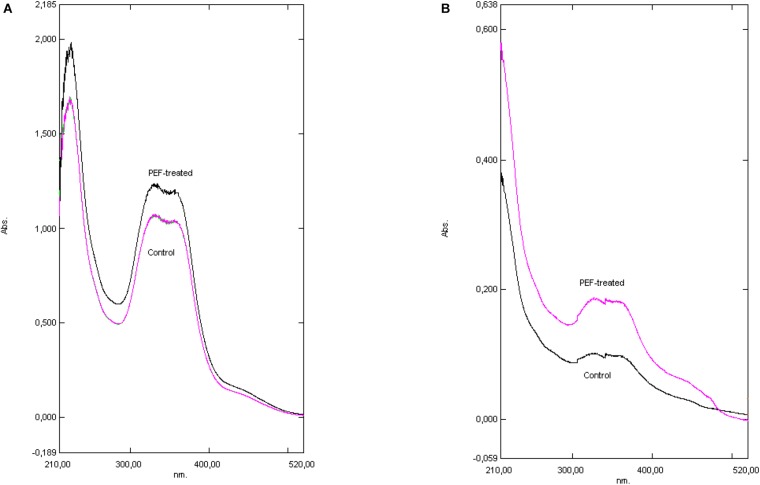
The effect of extraction media. Basic methanolic **(A)** versus Water **(B)**. For the quantification of the acids’ absorbance at 275, 325, and 355 nm was used. **(A)** The UV–vis spectrum of both samples, PEF treated and Control (bitter hop was suspended in methanol). **(B)** The UV–vis spectrum of both samples, PEF treated and Control (bitter hop was suspended in water).

Hop pellets from the *H. lupulus* plant contain both α-acids (humulones) and β-acids (lupulones) as well as many other compounds that interfere in the UV–vis spectrum (Figures 2, 3). The isomerization of α-acids to iso-α-acids during boiling is a process which strongly influences the taste of beer. The iso-α-acids are responsible for the distinct bitterness of the taste. The positive effect of the PEF treatment lies in the increase in the extractability of the α-acids which are then isomerized into iso-α-acids. The method applied for visualizing the PEF effect is a three-component analysis (Egts et al., 2012; Figure 3). Therefore, in order to determine the impact of the samples of hops treated with PEF, a spectrophotometric plot was followed to quantify the acids α and β, as well as those of the third component (iso-α-acids, etc.).

The spectra obtained from UV–vis plot are shown in Figures 3A,B and the calculated results are presented in Table 2. The spectra presented refer to treatment of bitter hop in methanol and in water. The decrease of the absorbance is attributed to the acids’ low solubility in water; nevertheless, the shape of the curve is the same in both treatment media, where the hop exhibits a similar physical and chemical response.

The spectra obtained from the UV–vis plot are presented in Figures 3A,B and the calculated results are presented in Table 2. The spectra presented refer to the treatment of bitter hop pellets in methanol and in water. The decrease in absorbance is attributed to the low solubility of acids in water; however, the shape of the curve is the same in both processing media, where the hop pellets have a similar physical and chemical response.

According to the results shown in Table 2, the difference of the α-acids and β-acids regarding the bitter hop are ranked between 9.1 and 23.7%. More specifically, the α-acids of the PEF treated sample were 23.7% higher than those of the control displaying in this manner the positive aspects of this treatment. As it has already been mentioned, humulones are isomerized into iso-α-acids, while the β-acids (also 9.1% higher) are mostly oxidized rather than isomerized. It was also observed, by employing different extraction media (solvents) or different varieties of hop, the results exhibited significant deviations (Figure 2). The aromatic hop (low concentration of α-acids and β-acids), under the same experimental conditions, showed no differences in PEF treatment and control. Finally, in order to examine the significance of the absence of water in the dried hop, the hops were hydrated for 30 min before treatment. This process produced similar results but with lower concentration in acids in comparison with the sample that was not hydrated. The aforementioned results are summarized in Tables 2, 3. During this process, methanol was used as solvent with the purpose to evaluate the PEF treatment. The extractability in methanol is intermediate between non-polar solvents like toluene and polar solvents like water.

**TABLE 3:** Content (mg/L) in α-acids and β-acids of toluene extracted bitter hop with UV–vis analysis.

**Acids**	**Control**	**PEF treated**	**Difference**
α-acids	11.8	25.8	118.03%
β-acids	7.8	14.5	85.39%

### Capacity of PEF Treatment Chamber and the Effect of Extraction Media

When the processing chamber is filled with a liquid or solid element, it becomes a capacitor. The electrodes become the conductors and the sample, which is being processed, the dielectric. The higher the conductivity of the product, the easier the electrical current flows. Thus, for high conductivity samples, a lower voltage should be used to avoid sparks. The capacity in water is much higher than the electric capacity in solvents such as methanol or ethanol (Table 4).

**TABLE 4:** Capacity of PEF treatment chamber with different solvents.

**Sample**	**Capacitance (μF)**	**Water (mL)/methanol or ethanol (mL)/plant material (gr)**
Water suspended hop	56.0	50/0/2.5
Methanol with hydrated hop	27.9	25/25/2.5
Methanol with dried hop	14.9	0/50/2.5
Ethanol with hydrated hop	24.2	25/25/2.5
Ethanol with dried hop	54.0	0/50/2.5

Most studies in the literature that assess the composition and release of hop ingredients in wort have been carried out with solvents such as methanol or other non-polar solvents (pentane and toluene). During the production of beer, the extraction of the hop constituents takes place in an almost hydro environment. In view of this, this study was carried out using aqueous media combined with pure methanol. The hop pellets were hydrated with pure HPLC water, and then suspended in methanol or pure water before treatment with PEF. In all these environments, the capacity of the processing cell was measured.

The hydrated hop results (Figure 3B) showed a week absorbance after PEF treatments across all spectra. By comparing the percentage of α- and β-acids extracted from hydrated bitter hops, we can conclude that due to their insolubility, the concentration of humulones and lupulones was much lower for PEF and the control samples and, consequently, their absorbance showed lower values. However, by examining their differences in percentages (Table 2), it can be concluded that the relative extractability due to PEF in water of acids and other compounds is higher than in non-polar solvents.

### Time Influence

**FIGURE 4:**
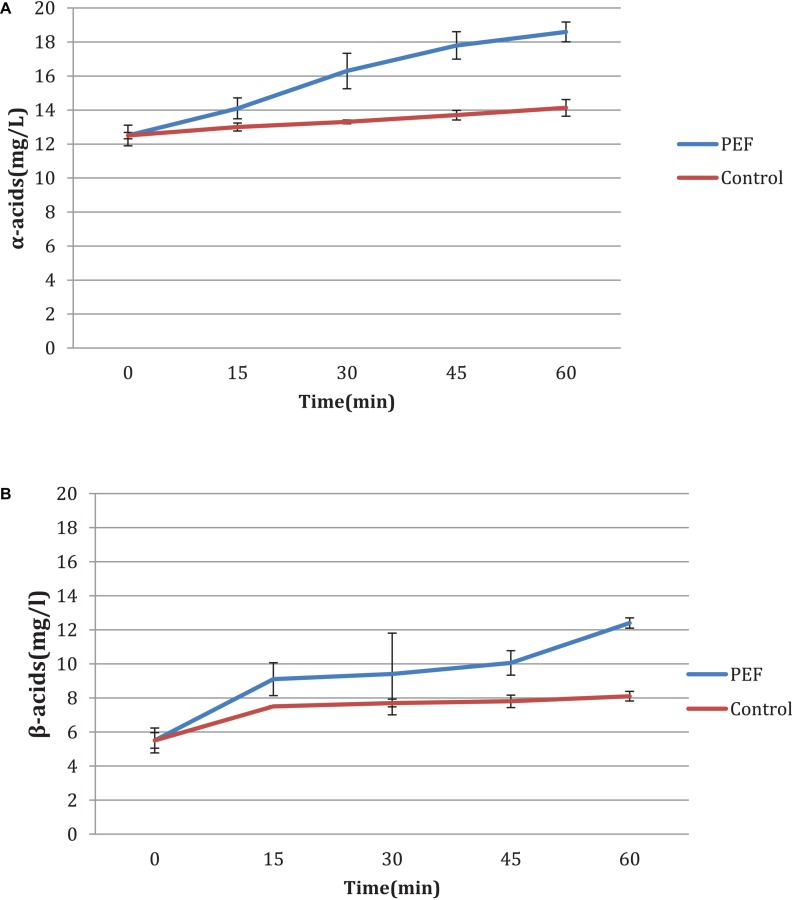
**(A,B)** Influence of time of treatment to bitter hop samples.

**FIGURE 5:**
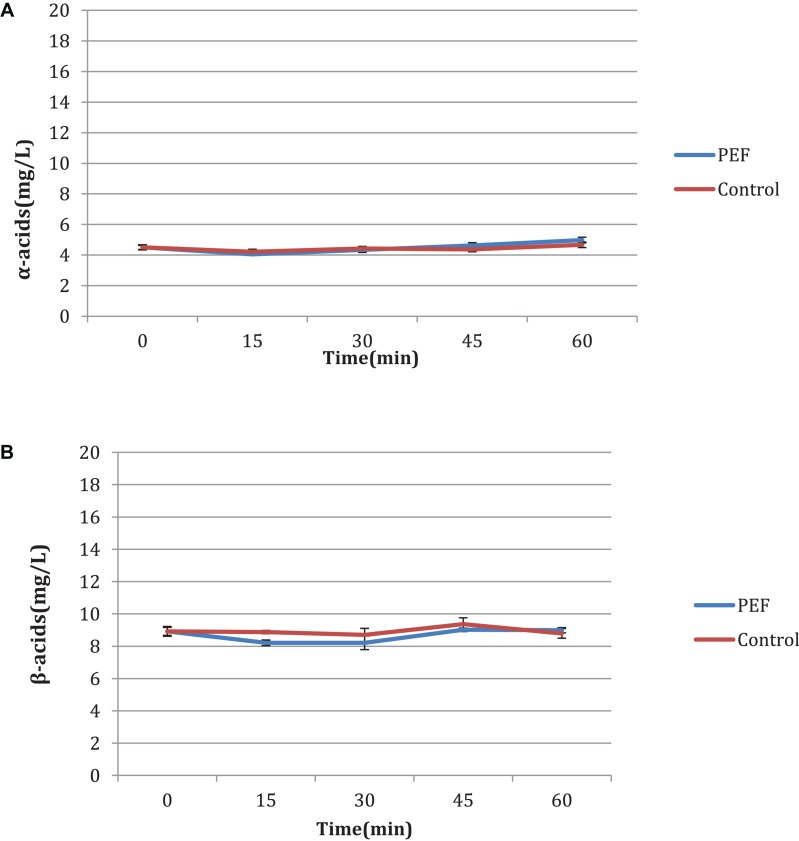
**(A,B)** Influence of time of treatment to aromatic hop samples.

An additional experiment was carried out to measure the influence of the duration of the PEF, as well as the differences between the two varieties, aromatic and bitter. It is observed that in the bitter, the acid concentration increases with the treatment time (Figures 4A,B) with or without PEF treatment. The variety of hops seems to have a significant effect on the final results. As shown in Figures 5A,B, the aromatic hops treated with PEF did not show any particular difference compared to the control. In addition, over time, the control samples and the samples treated with PEF have a negligible increase in the concentration of α and β-acids. We can deduce that in aromatic hops, the acids are mainly in free form unlike bitter, in which an amount of acids is probably localized in plant cells and is released after cell rupture with PEF.

### Volatile Analysis

The impact of the application of PEF in volatile compounds of bitter hops is presented in Table 5, while the average of the α and β-acids from the GC-MS analysis (compared to the spectrophotometric analysis) in Table 6. In order to avoid contamination of the GC column, an intermediate purification step was applied, using a solid phase extraction column (SPE). This purification step was carried out in order to avoid liquid-liquid extraction of the treated samples and thus avoid deterioration of the GC columns with waxes and non-volatile hop resins (Stevens, 1967; American Society of Brewing Chemists, 1992, Eri et al., 2000). The hops are rich in resins which are easily extracted by non-polar solvents and thus deteriorate the GC columns during analysis.

**TABLE 5:** Volatile analysis (mg/L) of bitter hop with or without PEF treatment.

**Compound**	**Pef treated**		**Control**	
	**Average**	**S.D.**	**Average**	**S.D.**
β-Myrcene	0.083	0.052	0.092	0.003
2-octanol	0.125	0.000	0.125	0.000
Caryophyllene	0.290	0.175	0.266	0.070
β-Cubebene	0.019	0.009	n.d.	
Humulene	0.943	0.578	0.889	0.203
γ-Muurolene	0.023	0.015	0.022	0.019
γ-Cadinene	0.032	0.004	0.023	0.006
β-Cadinene	0.008	0.002	n.d.	
α-Cadinene	0.008	0.005	0.008	0.003
δ-Cadinene	0.069	0.040	n.d.	
Geranyl isobutyrate	n.d.*		0.034	0.019
Hexadecane	0.036	0.008	0.023	0.002
Humulene epoxide 2	n.d.		0.025	0.006
Hexadecanoic acid methyl ester	0.053	0.021	0.047	0.010
Dehydro-cohumulinic acid or 3-Hydroxy-2-isobutyryl-5-(3-methyl-2-butenyl)-2,4-cyclopentadien-1-one	0.069	0.033	0.335	0.277
3-hydroxy-2-(1-hydroxy-3-methylbutylidene)-5-(3-methyl-2-butenylidene)-3-Cyclopenten-1-one	0.510	0.159	1.754	1.333
Linoleic acid methyl ester	0.036	0.019	0.032	0.006
Humulone	0.498	0.130	0.837	0.045
Isohumulone	5.977	1.134	4.325	0.275
Lupulone	16.630	2.498	11.269	0.926

*n.d. = Not detected.

**TABLE 6:** Averages (mg/L) of α- and β-acids of bitter hop PEF treated and control samples extracted with methanol.

**Method**	**Acids**	**Pef Treated**		**Control**		**Increase (%)**
			
		**Average**	**S.D.**	**Average**	**S.D.**	
GC-MS	α-acids	6.4	1.0	5.2	0.2	25.45
	β-acids	16.6	2.5	11.3	0.9	47.56
UV–vis	α-acids	16.2	1.0	13.1	0.1	23.66
	β-acids	9.6	2.4	8.8	0.2	9.09

Myrcene, β-caryophyllene and α-humulene are the most abundant terpenes in hops. In dry granules, their aroma is persistent and characteristic. In beer, they are lost as odor descriptors. They are also insoluble in wort and beer but their oxidation leads to derivative compounds, such as their epoxides (for example humulene epoxide) or humulol alcohols, which appear in the final product depending on the time of adding hops.

As previously mentioned, sesquiterpenes such as humulene, caryophyllene and β-pinene (oxidation product of myrcene) are the main constituents of essential oil of fresh hops. More specifically, it is evident (Table 5) that the application of PEF has a small but significant increase in the concentration of these compounds. In particular, in the control samples, the humulene, caryophyllene and β-pinene had a concentration of 0.89, 0.27, and 0.09 mg/L, while in the treated samples at PEF, there was an increase of 0.94, 0.29, and 0.08 mg/L, respectively. The application of PEF had a limited influence on the concentration of these volatile compounds and mainly in the monoterpens. The oxygenated fractions of the hop aroma (Deinzer and Yang, 1994) can be synergistic by contributing to the “hops” of beer (Siebert, 1994). All of these compounds have an active flavor in beer with very low flavor thresholds (ppb) and depending on when they are added, they play an important role in the character of hops (Preis and Mitter, 1995). From this point of view, even a slight increase in terpene precursors (humulene, caryophyllene and myrcene) is important for the final hoppy taste of beer.

**FIGURE 6:**
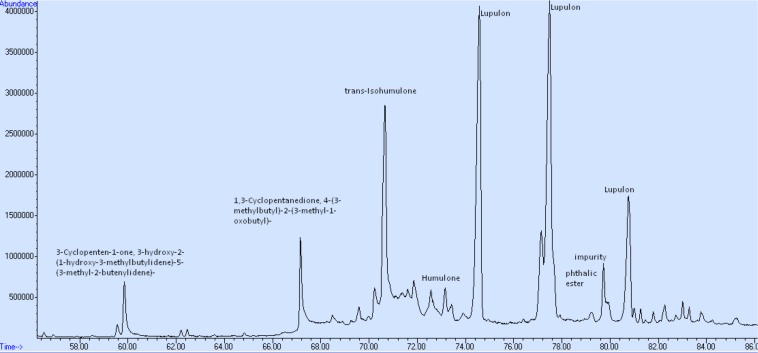
GC-MS analysis after purification thought SPE.

**FIGURE 7:**
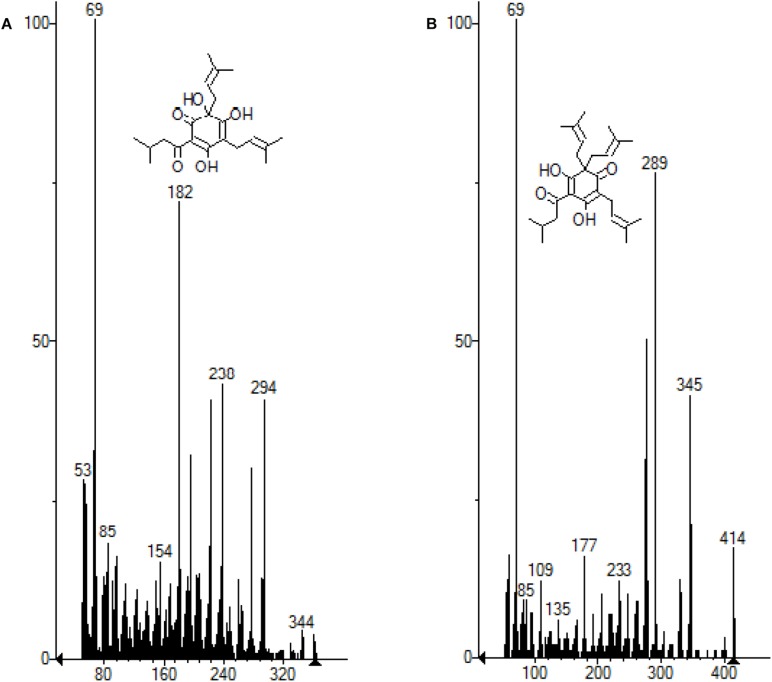
**(A)** Molecule of the humulone subtracted from the MS chromatogram. **(B)** Molecule of lupulon subtracted from the MS chromatogram.

Liquid-liquid extraction involves a heating step which can degrade the initial profile of volatiles. The SPE method used before the GC-MS analysis allowed a clear separation of the compounds and a “clean” chromatogram, as shown in Figure 6. The molecules of the main acids (humulone and lupulone) of the MS chromatogram are presented in the Figures 7A,B. Table 5 shows the effect of PEF on the (average) concentration of α and β-acids in bitter hop varieties. The concentration of acids should be higher in bitter hops. The extraction of α and β-acids soluble in methanol also increases with the application of PEF, with the intensity of the electric field and with the extension of the duration of the treatment.

As indicated in the samples treated with PEF, the bitter acids in the sample had a concentration 40.66% higher than that of the control. More specifically, the samples treated with PEF had a higher concentration compared to the control (25.45 and 47.56% of α and β-acids, respectively). This result is of capital importance since the aim of bitter hops is to strengthen bitterness.

### DPPH^∙^

The treated extracts maintained their antiradical activity (Table 7) and, in the case of extracted methanol, an increase of about 10% was observed. The water treated samples demonstrated almost the same antiradical activity. The low water extractability probably does not allow the proper evaluation of the results.

**TABLE 7:** Percentage of inhibition of DPPH^∙^ radical (I%) of hop extracts.

**Sample**	***I%***	
	**PEF treated**	**Control**
Hydrated (bitter)	70.27	69.89
Methanol (bitter)	73.19	83.35
Methanol (aromatic)	82.47	81.32

## Conclusion

In conclusion, this study aimed to extract α-acids and β-acids of two different varieties of hops using PEF. During these experiments, different solvents and different methods of analysis were used. According to the results, samples of hops treated with PEF showed higher concentrations of humulones and lupulones (the main representatives of α-acids and β-acids, respectively). PEF conditions (1.5 kV/cm; 15 μs and 1800 pulses) increased the total bitter acids (a + β) and sesquiterpenes extraction from bitter hop approximatively by 1.3 times. The PEF treatment enhanced the extraction of α-acids from 21 to 100% and from 9 to 120% for β-acids. The amount of extracted acids was a function of the solvent and the time of treatment. PEF treatment of hop pellets did not cause any substantial changes in HSI that would indicate possible further degradation. Hops maintained their antiradical activity, which, in some cases, was increased. Therefore, it can be concluded that the extraction of α- and β-acids was enhanced by PEF application and should be further investigated in order to optimize their concentration by utilizing water base solvents or by minimizing the time of the PEF treatment in pilot plant conditions before industrial applications.

## Data Availability Statement

All datasets generated for this study are included in the article/supplementary material.

## Author Contributions

GN: conception and execution of the PEF work. ET: analysis of GC-MS. FD: analysis of hop and antioxidant properties. EB: writing – review and editing. SL, PT, and VD: materials and methods setup.

## Conflict of Interest

The authors declare that the research was conducted in the absence of any commercial or financial relationships that could be construed as a potential conflict of interest.
